# Clarithromycin monohydrate: a synchrotron X-ray powder study

**DOI:** 10.1107/S1600536812005090

**Published:** 2012-02-10

**Authors:** Shuji Noguchi, Sadahiro Fujiki, Yasunori Iwao, Keiko Miura, Shigeru Itai

**Affiliations:** a Graduate School of Pharmaceutical Sciences, University of Shizuoka, 52-1 Yada, Suruga-ku, Shizuoka 422-8526, Japan; b Industrial Application Division, Japan Synchrotron Radiation Research Institute, 1-1-1 Kouto, Sayo-gun, Hyogo 679-5198, Japan

## Abstract

In the crystal structure of the title compound, clarithromycin (CAM) monohydrate, C_38_H_69_NO_13_·H_2_O, the water mol­ecule behaves as a proton donor and is hydrogen bonded to the hy­droxy O atom of the CAM cladinose ring. The hy­droxy O atom also behaves as a proton donor, forming an inter­molecular hydrogen bond with one of the hy­droxy groups of the 14-membered aglycone ring. The CAM mol­ecules are linked through these hydrogen bonds into chains running parallel to the *c* axis.

## Related literature

For background to the title compound, see Avrutov *et al.* (2003 ▶); Noguchi, Fujiki *et al.* (2012 ▶). For information relating to the pharmaceutical properties of CAM, see: Yajima *et al.* (1999 ▶, 2002 ▶); Fujiki *et al.* (2011 ▶); Liu *et al.* (1999 ▶). For related structures, see: Noguchi, Miura *et al.* (2012 ▶; form I, anhydrate); Jin *et al.* (2011 ▶; form 0, ethanol solvate); Stephenson *et al.* (1997 ▶; form II, anhydrate); Liang & Yao (2008 ▶; form III, acetonitrile solvate); Parvez *et al.* (2000 ▶; hydro­chloride salt); Iwasaki *et al.* (1993 ▶; methanol solvate).
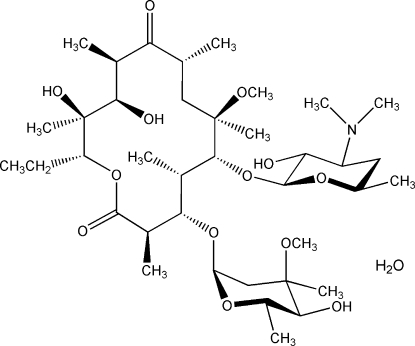


## Experimental

### 

#### Crystal data

C_38_H_69_NO_13_·H_2_O
*M* *_r_* = 765.97
Orthorhombic, 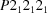

*a* = 15.6999 (2) Å
*b* = 18.8817 (2) Å
*c* = 15.0267 (2) Å
*V* = 4454.53 (9) Å^3^
*Z* = 4
Synchrotron radiation, λ = 1.3000 Å
μ = 0.41 mm^−1^
*T* = 298 K
cylinder, 3.0 × 0.3 mm

#### Data collection

BL-19B2 Debye–Scherrer camera diffractometer
Specimen mounting: capilary
Data collection mode: transmission
Scan method: Stationary detector
2θ_fixed_ = 65

#### Refinement

*R*_p_ = 0.038
*R*_wp_ = 0.052
*R*_exp_ = 0.016
*R*_Bragg_ = 0.059
*R*(*F*) = 0.076
*R*(*F* ^2^) = 0.07617
χ^2^ = 11.020
6201 data points
188 parameters
96 restraints
H-atom parameters not refined

### 

Data collection: local software (Osaka *et al.*, 2010 ▶; Takata *et al.*, 2002 ▶); cell refinement: *EXPO2009* (Altomare *et al.*, 2009 ▶) and *RIETAN-FP* (Izumi & Momma, 2007 ▶); data reduction: local software (Takata *et al.*, 2002 ▶); program(s) used to solve structure: *CCP4* (Collaborative Computational Project, Number 4, 1994 ▶); program(s) used to refine structure: *CCP4*, *RIETAN-FP* and *Jmol* (Hanson, 2010 ▶); molecular graphics: *CCP4MG* (McNicholas *et al.*, 2011 ▶); software used to prepare material for publication: *publCIF* (Westrip, 2010 ▶).

## Supplementary Material

Crystal structure: contains datablock(s) global, I. DOI: 10.1107/S1600536812005090/hb6588sup1.cif

Rietveld powder data: contains datablock(s) I. DOI: 10.1107/S1600536812005090/hb6588Isup2.rtv

Supplementary material file. DOI: 10.1107/S1600536812005090/hb6588Isup3.cml

Additional supplementary materials:  crystallographic information; 3D view; checkCIF report

## Figures and Tables

**Table 1:** Hydrogen-bond geometry (Å, °)

*D*—H⋯*A*	*D*—H	H⋯*A*	*D*⋯*A*	*D*—H⋯*A*
O6—H67⋯O7	0.83	2.30	2.68 (3)	108
O7—H68⋯O8	0.82	2.13	2.83 (3)	143
O12—H69⋯O11	0.83	2.34	2.75 (4)	111
O6—H67⋯O12^i^	0.83	2.39	2.73 (3)	105
O12—H69⋯O6^ii^	0.83	2.43	2.73 (3)	102
O14—H70⋯O12	0.97	1.70	2.65 (4)	168
O14—H71⋯O7^ii^	0.95	2.58	3.51 (4)	166

Symmetry codes: (i) 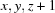; (ii) 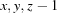.

**Fig. 1.**
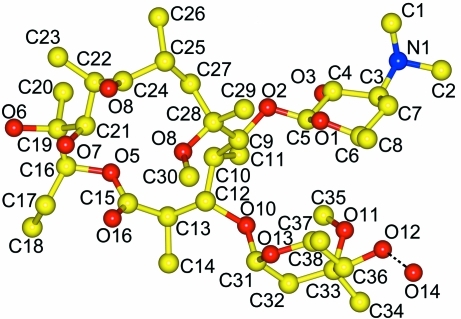
The molecular structure of CAM with atoms represented as spheres of arbitrary radii. C, N, and O atoms are shown in yellow, blue and red, respectively. H atoms have been omitted for clarity. Hydrogen bonding between CAM and a water molecule is indicated by a dashed line.

**Fig. 2.**
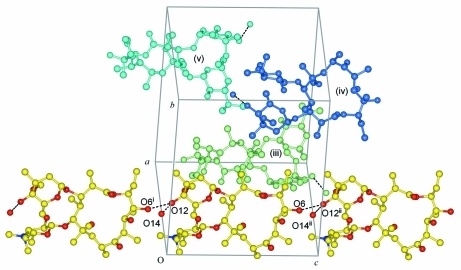
Packing view of CAM. The molecular chains generated by hydrogen bonding between CAM molecules along the c axis are coloured as in Fig. 1. [Symmetry code: (i) x, y, z - 1, (ii) x, y, z + 1.] Molecules of symmetry codes (iii) x + 1/2, -y + 1/2, -z + 1, (iv) -x + 1/2, -y + 1, z + 1/2, and (v) -x + 1, y + 1/2, -z + 1/2 are shown in light green, light blue and cyan, respectively.

**Fig. 3.**
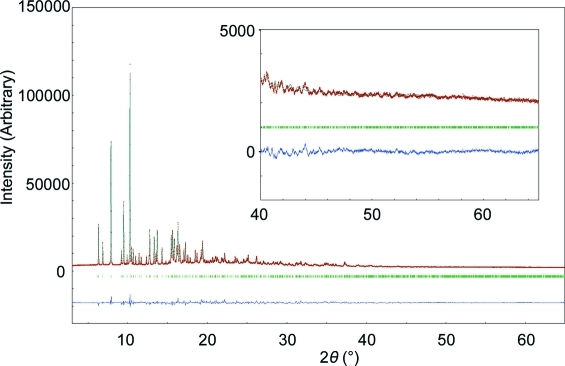
The final Rietveld plot. The experimental diffraction profile is indicated by red crosses. The calculated diffraction and difference profiles are depicted as solid green and blue lines, respectively. The vertical green bars correspond to the positions of the Bragg peaks.

## supplementary crystallographic information

### Comment

CAM is a macrolide antibiotic containing a 14-membered ring. Current clinical formulations of CAM use crystals of the stable anhydrate form II in the treatment of infections caused by bacteria (Yajima *et al.*, 1999, 2002; Fujiki *et al.*, 2011). Another anhydrate crystal form of CAM, metastable form I, has a dissolution rate three times greater than that of form II (Liu *et al.*, 1999), indicating its potential use for a new drug formulation. We recently found that CAM form I spontaneously transforms to CAM monohydrate form IV when stored under high-humidity conditions at room temperature (Noguchi, Fujiki *et al.*, 2012). Although existence of form IV has been documented in the literature (Avrutov *et al.*, 2003), its structure remains unknown. As form IV is believed to be a possible impurity of form I, crystallographic characterization of form IV is necessary to enable a new drug formulation using form I to progress into practical use. We report here the crystal structure of form IV as determined by synchrotron powder X-ray diffraction analysis. The asymmetric unit of form IV contains one CAM molecule and one water molecule. The O14 atom of the water molecule behaves as a proton donor and is hydrogen-bonded to the hydroxy O12 atom of the CAM cladinose ring. Furthermore, the hydroxy O12 atom acts as a proton acceptor, forming an intermolecular hydrogen bond with the hydroxy O6^i^ atom of CAM aglycone ring (symmetry code in Table 1). Through this intermolecular O6^i^—O12 hydrogen bonding interaction, CAM molecules are linked into chains running parallel to the *c* axis, as shown in Fig. 2.

### Experimental

Powders of CAM form I were prepared as described (Noguchi, Miura *et al.*, 2012) and were converted to form IV by storing at greater than 90% relative humidity overnight in a hermetic glass container at 297 K. Relative humidity was measured by digital hygrometer AD-5683 (A&D, Tokyo, Japan). The powders of form IV thus obtained were enclosed in a 0.3 mm Lindemann glass capillary. The powder diffraction data were collected at SPring-8 BL19B2 (Osaka *et al.*, 2010; Takata *et al.*, 2002). The sample was rotated at 1 r min^-1^ to reduce the possible preferential orientation and was kept at 298 K.

### Refinement

The determination of cell parameters and space group and the extraction of the Bragg peak intensities from the powder diffraction data were carried out using *EXPO2009*. The initial structure was determined by the molecular replacement method using *MOLREP* implemented in *CCP4*. The search model employed was form 0 of the CAM crystal structure (Jin *et al.*, 2011). All H atoms were excluded from the model and the isotropic atomic displacement parameters were fixed at a value of 0.089 Å^2^. Reflections between 12.1 and 2.50 Å *d*-spacings were used for the calculation. The structure solution of the molecular replacement was refined using *REFMAC* implemented in *CCP4*. The bond lengths and bond angles were restrained to those of the form 0 crystal structure. The crystallographic *R* factor converged at 0.245. In the difference Fourier map, the positive spherical density was found at a distance of approximately 2.7 Å from the hydroxy O12 atom of the CAM cladinose ring. The O atom of the water molecule was placed at this density and the model was further refined, resulting in the convergence of the *R* factor at 0.201. This partially refined structure provided the starting model for Rietveld refinement. The geometry of the CAM molecule was restrained as described above. H atoms were placed at their theoretical positions using *EXPO2009* and *Jmol* and were refined as riding. The overall atomic displacement parameter was applied to all atoms including H atoms, and was refined isotropically. The observed and Rietveld refined calculated powder patterns are shown in Fig. 3. The r.m.s differences of the bond lengths and angles from their target values were 0.023 Å and 2.7 °, respectively.

### Crystal data

**Table:**

C_38_H_69_NO_13_·H_2_O	*F*(000) = 1672.00
*M**_r_* = 765.97	*D*_x_ = 1.142 Mg m^−^^3^
Orthorhombic, *P*2_1_2_1_2_1_	Synchrotron radiation, λ = 1.3000 Å
Hall symbol: P 2ac 2ab	µ = 0.41 mm^−^^1^
*a* = 15.6999 (2) Å	*T* = 298 K
*b* = 18.8817 (2) Å	Particle morphology: powder
*c* = 15.0267 (2) Å	white
*V* = 4454.53 (9) Å^3^	cylinder, 3.0 × 0.3 mm
*Z* = 4	Specimen preparation: Prepared at 298 K and 101 kPa

### Data collection

**Table:**

BL-19B2 Debye–Scherrer camera diffractometer	Data collection mode: transmission
Radiation source: synchrotron, SPring-8 BL19B2	Scan method: Stationary detector
Si(111) monochromator	2θ_fixed_ = 65
Specimen mounting: capilary	

### Refinement

**Table:**

Least-squares matrix: selected elements only	Profile function: split pseudo-Voigt function
*R*_p_ = 0.038	188 parameters
*R*_wp_ = 0.052	96 restraints
*R*_exp_ = 0.016	0 constraints
*R*_Bragg_ = 0.059	H-atom parameters not refined
*R*(*F*) = 0.076	Weighting scheme based on measured s.u.'s 1/[Y_i_]
*R*(*F*^2^) = 0.07617	(Δ/σ)_max_ = 0.011
χ^2^ = 11.020	Background function: Legendre polynomials
6201 data points	

### Fractional atomic coordinates and isotropic or equivalent isotropic displacement parameters (Å^2^)

**Table:**

	*x*	*y*	*z*	*U*_iso_*/*U*_eq_	
O1	0.024 (1)	0.1102 (8)	0.205 (1)	0.066 (5)*	
C1	0.154 (2)	−0.074 (2)	0.101 (2)	0.066 (5)*	
O2	0.108 (2)	0.1127 (9)	0.327 (1)	0.066 (5)*	
C2	0.217 (3)	−0.007 (2)	−0.026 (1)	0.066 (5)*	
O3	0.256 (1)	0.079 (1)	0.220 (2)	0.066 (5)*	
C3	0.156 (1)	0.0568 (7)	0.095 (1)	0.066 (5)*	
O4	0.051 (1)	0.356 (1)	0.609 (1)	0.066 (5)*	
C4	0.168 (1)	0.065 (1)	0.197 (1)	0.066 (5)*	
O5	0.166 (1)	0.286 (1)	0.638 (1)	0.066 (5)*	
C5	0.107 (1)	0.119 (1)	0.236 (1)	0.066 (5)*	
O6	0.089 (1)	0.239 (1)	0.8636 (9)	0.066 (5)*	
C6	0.0191 (8)	0.1221 (7)	0.111 (1)	0.066 (5)*	
O7	−0.0338 (9)	0.2408 (9)	0.740 (2)	0.066 (5)*	
C7	0.064 (1)	0.062 (1)	0.068 (1)	0.066 (5)*	
O8	−0.1207 (9)	0.122 (1)	0.670 (1)	0.066 (5)*	
C8	−0.073 (1)	0.117 (1)	0.092 (2)	0.066 (5)*	
O9	−0.032 (2)	0.210 (1)	0.484 (2)	0.066 (5)*	
C9	0.079 (1)	0.176 (1)	0.374 (1)	0.066 (5)*	
C10	0.150 (1)	0.221 (1)	0.422 (1)	0.066 (5)*	
C11	0.236 (1)	0.213 (1)	0.372 (2)	0.066 (5)*	
C12	0.121 (1)	0.297 (1)	0.419 (1)	0.066 (5)*	
C13	0.165 (1)	0.341 (1)	0.495 (1)	0.066 (5)*	
C14	0.170 (2)	0.420 (1)	0.473 (2)	0.066 (5)*	
C15	0.118 (1)	0.329 (1)	0.586 (1)	0.066 (5)*	
C16	0.1654 (9)	0.2915 (7)	0.737 (1)	0.066 (5)*	
C17	0.133 (1)	0.3633 (7)	0.763 (1)	0.066 (5)*	
C18	0.207 (2)	0.411 (1)	0.784 (3)	0.066 (5)*	
C19	0.1174 (8)	0.2269 (6)	0.7706 (8)	0.066 (5)*	
C20	0.179 (1)	0.1663 (7)	0.777 (2)	0.066 (5)*	
C21	0.0398 (9)	0.205 (1)	0.709 (1)	0.066 (5)*	
C22	0.0214 (9)	0.1267 (8)	0.708 (2)	0.066 (5)*	
C23	−0.005 (2)	0.095 (2)	0.797 (2)	0.066 (5)*	
C24	−0.0497 (8)	0.109 (1)	0.645 (1)	0.066 (5)*	
C25	−0.028 (1)	0.0617 (8)	0.564 (1)	0.066 (5)*	
C26	0.012 (2)	−0.008 (1)	0.599 (2)	0.066 (5)*	
C27	0.036 (1)	0.095 (1)	0.502 (2)	0.066 (5)*	
C28	0.003 (2)	0.1505 (9)	0.434 (1)	0.066 (5)*	
C29	−0.060 (1)	0.113 (2)	0.371 (2)	0.066 (5)*	
N1	0.198 (1)	−0.0086 (9)	0.071 (1)	0.066 (5)*	
C30	−0.108 (2)	0.245 (2)	0.457 (3)	0.066 (5)*	
C31	0.096 (1)	0.3798 (7)	0.282 (1)	0.066 (5)*	
C32	0.147 (2)	0.4097 (8)	0.204 (2)	0.066 (5)*	
C33	0.144 (2)	0.3696 (9)	0.113 (2)	0.066 (5)*	
C34	0.181 (3)	0.418 (2)	0.044 (2)	0.066 (5)*	
C35	0.259 (2)	0.288 (1)	0.164 (2)	0.066 (5)*	
C36	0.050 (1)	0.355 (2)	0.089 (2)	0.066 (5)*	
C37	0.0039 (9)	0.319 (2)	0.166 (2)	0.066 (5)*	
C38	−0.088 (1)	0.309 (2)	0.147 (2)	0.066 (5)*	
O10	0.148 (1)	0.326 (1)	0.322 (1)	0.066 (5)*	
O11	0.189 (1)	0.301 (1)	0.109 (2)	0.066 (5)*	
O12	0.040 (2)	0.310 (1)	0.013 (2)	0.066 (5)*	
O13	0.013 (1)	0.357 (1)	0.251 (1)	0.066 (5)*	
O14	−0.108 (2)	0.328 (1)	−0.069 (2)	0.066 (5)*	
H1	0.18752	−0.11445	0.08465	0.066*	
H2	0.14803	−0.0724	0.16457	0.066*	
H3	0.09936	−0.07653	0.07354	0.066*	
H4	0.16522	−0.00535	−0.0587	0.066*	
H5	0.25191	0.03289	−0.03953	0.066*	
H6	0.24771	−0.05005	−0.04139	0.066*	
H7	0.18159	0.09522	0.06236	0.066*	
H8	0.15338	0.02044	0.2234	0.066*	
H9	0.12561	0.16482	0.21795	0.066*	
H10	0.04357	0.16609	0.09204	0.066*	
H11	0.06159	0.06938	0.00406	0.066*	
H12	0.03573	0.01907	0.08252	0.066*	
H13	−0.10242	0.15481	0.12278	0.066*	
H14	−0.08217	0.12197	0.02935	0.066*	
H15	−0.0944	0.07245	0.11207	0.066*	
H16	0.05924	0.21174	0.33347	0.066*	
H17	0.15866	0.20417	0.48194	0.066*	
H18	0.22516	0.21423	0.30905	0.066*	
H19	0.27191	0.25247	0.38771	0.066*	
H20	0.26242	0.16985	0.38811	0.066*	
H21	0.0607	0.30225	0.42837	0.066*	
H22	0.22166	0.32306	0.50104	0.066*	
H23	0.11338	0.43968	0.46883	0.066*	
H24	0.20102	0.4445	0.51848	0.066*	
H25	0.19835	0.42629	0.41672	0.066*	
H26	0.22007	0.28954	0.76484	0.066*	
H27	0.10026	0.38318	0.71559	0.066*	
H28	0.09815	0.35908	0.81542	0.066*	
H29	0.23905	0.41951	0.73017	0.066*	
H30	0.18542	0.45621	0.8051	0.066*	
H31	0.24274	0.39006	0.82833	0.066*	
H32	0.14917	0.12451	0.79505	0.066*	
H33	0.20586	0.15915	0.72105	0.066*	
H34	0.22166	0.17769	0.8214	0.066*	
H35	0.05522	0.21868	0.64964	0.066*	
H36	0.07503	0.10624	0.6907	0.066*	
H37	−0.06312	0.10794	0.80989	0.066*	
H38	0.00025	0.0447	0.79565	0.066*	
H39	0.03108	0.11387	0.8437	0.066*	
H40	−0.08013	0.05386	0.53219	0.066*	
H41	−0.02796	−0.03231	0.6374	0.066*	
H42	0.02733	−0.03813	0.55089	0.066*	
H43	0.06242	0.00286	0.63387	0.066*	
H44	0.07815	0.118	0.53798	0.066*	
H45	0.06197	0.05736	0.4687	0.066*	
H46	−0.08548	0.14665	0.33188	0.066*	
H47	−0.03038	0.07754	0.33667	0.066*	
H48	−0.10369	0.09003	0.40568	0.066*	
H49	−0.14675	0.21036	0.43356	0.066*	
H50	−0.1328	0.26788	0.5073	0.066*	
H51	−0.09414	0.27884	0.41213	0.066*	
H52	0.08197	0.41389	0.32742	0.066*	
H53	0.12529	0.457	0.19385	0.066*	
H54	0.20452	0.41209	0.22314	0.066*	
H55	0.24159	0.42459	0.05703	0.066*	
H56	0.17631	0.39594	−0.01318	0.066*	
H57	0.15325	0.46267	0.04479	0.066*	
H58	0.24994	0.30659	0.22227	0.066*	
H59	0.27045	0.23838	0.1669	0.066*	
H60	0.30803	0.31141	0.13802	0.066*	
H61	0.02618	0.40102	0.07467	0.066*	
H62	0.03089	0.27317	0.17169	0.066*	
H63	−0.09586	0.27116	0.10468	0.066*	
H64	−0.11828	0.29764	0.20071	0.066*	
H65	−0.11127	0.35198	0.12218	0.066*	
H66	0.28701	0.06779	0.17828	0.066*	
H67	0.04599	0.26422	0.86513	0.066*	
H68	−0.07662	0.21661	0.73170	0.066*	
H69	0.07204	0.27540	0.01797	0.066*	
H70	−0.05854	0.31967	−0.03228	0.066*	
H71	−0.09198	0.31147	−0.12651	0.066*	

### Geometric parameters (Å, º)

**Table:**

O1—C5	1.39 (2)	C5—H9	0.96
O1—C6	1.43 (2)	O6—H67	0.82
C1—N1	1.49 (4)	C6—H10	0.96
O2—C5	1.37 (2)	O7—H68	0.82
O2—C9	1.46 (3)	C7—H12	0.96
C2—N1	1.49 (2)	C7—H11	0.96
O3—C4	1.45 (2)	C8—H14	0.96
C3—N1	1.45 (2)	C8—H13	0.96
C3—C7	1.50 (2)	C8—H15	0.96
C3—C4	1.55 (2)	C9—H16	0.96
O4—C15	1.22 (2)	C10—H17	0.96
C4—C5	1.52 (2)	C11—H20	0.96
O5—C15	1.36 (2)	C11—H19	0.96
O5—C16	1.49 (2)	C11—H18	0.96
O6—C19	1.485 (18)	C12—H21	0.96
C6—C8	1.48 (2)	C13—H22	0.96
C6—C7	1.48 (2)	C14—H25	0.96
O7—C21	1.42 (2)	C14—H24	0.96
O8—C24	1.20 (2)	C14—H23	0.96
O9—C30	1.42 (4)	C16—H26	0.96
O9—C28	1.46 (3)	C17—H27	0.96
C9—C28	1.57 (3)	C17—H28	0.96
C9—C10	1.58 (2)	C18—H31	0.96
C10—C12	1.51 (3)	C18—H30	0.96
C10—C11	1.55 (3)	C18—H29	0.96
C12—C13	1.57 (2)	C20—H33	0.96
C13—C14	1.53 (3)	C20—H32	0.96
C13—C15	1.57 (2)	C20—H34	0.96
C16—C17	1.500 (19)	C21—H35	0.96
C16—C19	1.520 (18)	C22—H36	0.96
C17—C18	1.50 (3)	C23—H38	0.96
C19—C20	1.501 (19)	C23—H37	0.96
C19—C21	1.585 (19)	C23—H39	0.96
C21—C22	1.51 (2)	C25—H40	0.96
C22—C24	1.50 (3)	C26—H42	0.96
C22—C23	1.52 (4)	C26—H43	0.96
C24—C25	1.55 (2)	C26—H41	0.96
C25—C27	1.51 (3)	C27—H44	0.96
C25—C26	1.55 (3)	C27—H45	0.96
C27—C28	1.55 (3)	C29—H46	0.96
C28—C29	1.54 (4)	C29—H47	0.96
C31—O10	1.44 (2)	C29—H48	0.96
C31—O13	1.45 (2)	C30—H50	0.96
C31—C32	1.53 (3)	C30—H51	0.96
C32—C33	1.56 (4)	C30—H49	0.96
C33—O11	1.48 (3)	C31—H52	0.96
C33—C34	1.50 (4)	C32—H54	0.96
C33—C36	1.54 (4)	C32—H53	0.96
C35—O11	1.40 (4)	C34—H57	0.96
C36—O12	1.43 (4)	C34—H56	0.96
C36—C37	1.53 (4)	C34—H55	0.98
C37—O13	1.47 (4)	C35—H58	0.96
C37—C38	1.48 (2)	C35—H59	0.96
C1—H1	0.96	C35—H60	0.96
C1—H3	0.96	C36—H61	0.96
C1—H2	0.96	C37—H62	0.96
C2—H4	0.96	C38—H64	0.96
C2—H5	0.96	C38—H65	0.96
C2—H6	0.96	C38—H63	0.96
O3—H66	0.82	O12—H69	0.82
C3—H7	0.96	O14—H70	0.96
C4—H8	0.96	O14—H71	0.96
			
C5—O1—C6	111.2 (13)	H15—C8—C6	109.3
C5—O2—C9	114.0 (16)	C28—O9—H51	114.2
N1—C3—C7	115.3 (13)	H16—C9—O2	111.4
N1—C3—C4	106.0 (13)	H16—C9—C28	109.9
C7—C3—C4	112.1 (13)	H17—C10—C12	112.2
O3—C4—C5	112.8 (16)	H17—C10—C11	107.7
O3—C4—C3	111.7 (16)	H17—C10—C9	111.0
C5—C4—C3	111.8 (13)	C11—C10—H16	107.1
C15—O5—C16	122.0 (15)	H20—C11—H19	109.8
O2—C5—O1	109.5 (18)	H20—C11—H18	111.0
O2—C5—C4	108.6 (16)	H20—C11—C10	109.8
O1—C5—C4	112.4 (14)	H19—C11—H18	109.0
O1—C6—C8	103.5 (16)	H19—C11—C10	109.0
O1—C6—C7	106.5 (12)	H18—C11—C10	109.3
C8—C6—C7	109.3 (14)	H21—C12—C10	112.4
C6—C7—C3	112.9 (13)	H21—C12—C13	106.1
C30—O9—C28	122 (3)	H21—C12—O10	110.8
O2—C9—C28	105.3 (16)	H22—C13—C14	109.0
O2—C9—C10	116.2 (17)	H22—C13—C15	108.2
C28—C9—C10	116.1 (13)	H22—C13—C12	107.1
C12—C10—C11	109.9 (14)	H25—C14—H24	110.0
C12—C10—C9	106.6 (13)	H25—C14—H23	109.2
C11—C10—C9	109.9 (14)	H25—C14—C13	109.6
C10—C12—C13	110.4 (13)	H24—C14—H23	109.1
C10—C12—O10	105.7 (13)	H24—C14—C13	109.5
C13—C12—O10	111.2 (14)	H23—C14—C13	109.3
C14—C13—C15	110.7 (17)	H26—C16—O5	115.5
C14—C13—C12	112.4 (16)	H26—C16—C17	102.6
C15—C13—C12	110.5 (13)	H26—C16—C19	105.4
O4—C15—O5	124.5 (16)	H27—C17—H28	109.1
O4—C15—C13	126.3 (16)	H27—C17—C18	109.7
O5—C15—C13	109.1 (14)	H27—C17—C16	109.9
O5—C16—C17	109.0 (13)	H28—C17—C18	108.6
O5—C16—C19	106.2 (12)	H28—C17—C16	109.4
C17—C16—C19	118.1 (12)	H28—C17—H26	109.2
C18—C17—C16	109.5 (15)	H31—C18—H30	110.0
O6—C19—C20	104.5 (15)	H31—C18—H29	109.6
O6—C19—C16	109.8 (12)	H31—C18—C17	110.2
O6—C19—C21	111.0 (11)	H30—C18—H29	108.5
C20—C19—C16	108.3 (11)	H30—C18—C17	109.6
C20—C19—C21	109.5 (13)	H29—C18—C17	108.9
C16—C19—C21	113.4 (11)	C16—C19—H67	104.0
O7—C21—C22	108.4 (13)	H33—C20—H32	110.3
O7—C21—C19	108.1 (15)	H33—C20—H34	109.5
C22—C21—C19	114.2 (14)	H33—C20—C19	109.5
C24—C22—C21	111.6 (16)	H32—C20—H34	109.3
C24—C22—C23	105.3 (16)	H32—C20—C19	109.4
C21—C22—C23	115 (2)	H34—C20—C19	108.9
O8—C24—C22	116.6 (16)	H35—C21—O7	113.0
O8—C24—C25	124.6 (15)	H35—C21—C22	107.0
C22—C24—C25	117.4 (13)	H35—C21—C19	106.5
C27—C25—C24	113.1 (14)	H35—C21—H68	112.6
C27—C25—C26	107.1 (16)	H68—C21—H36	113.9
C24—C25—C26	108.2 (15)	H36—C22—C24	113.0
C25—C27—C28	117.8 (16)	H36—C22—C21	103.2
O9—C28—C29	115 (3)	H36—C22—C23	108.8
O9—C28—C27	107.9 (17)	H38—C23—H37	110.1
O9—C28—C9	110.2 (17)	H38—C23—H39	109.5
C29—C28—C27	108.0 (19)	H38—C23—C22	109.8
C29—C28—C9	106.0 (14)	H37—C23—H39	109.0
C27—C28—C9	109 (2)	H37—C23—C22	109.3
C3—N1—C2	108.6 (19)	H39—C23—C22	109.0
C3—N1—C1	115.0 (18)	H40—C25—C27	108.6
C2—N1—C1	114 (2)	H40—C25—C24	107.1
O10—C31—O13	115.8 (14)	H40—C25—C26	112.5
O10—C31—C32	106.6 (15)	H42—C26—H43	110.0
O13—C31—C32	109.5 (16)	H42—C26—H41	109.9
C31—C32—C33	118.4 (17)	H42—C26—C25	110.5
O11—C33—C34	109 (3)	H43—C26—H41	108.1
O11—C33—C36	107 (2)	H43—C26—C25	109.0
O11—C33—C32	117 (2)	H41—C26—C25	109.3
C34—C33—C36	109 (3)	H44—C27—H45	109.4
C34—C33—C32	107.3 (19)	H44—C27—C25	107.1
C36—C33—C32	109 (2)	H44—C27—C28	107.9
O12—C36—C37	107 (3)	H45—C27—C25	106.6
O12—C36—C33	113 (2)	H45—C27—C28	107.5
C37—C36—C33	111 (2)	H46—C29—H47	110.0
O13—C37—C38	109 (2)	H46—C29—H48	109.5
O13—C37—C36	113 (3)	H46—C29—C28	109.3
C38—C37—C36	112 (2)	H47—C29—H48	109.4
C31—O10—C12	118.0 (13)	H47—C29—C28	109.6
C35—O11—C33	120 (2)	H48—C29—C28	109.1
C31—O13—C37	120.7 (14)	H50—C30—H51	110.6
H1—C1—H3	110.1	H50—C30—H49	109.6
H1—C1—H2	109.6	H50—C30—O9	109.3
H1—C1—N1	109.5	H51—C30—H49	109.6
H3—C1—H2	109.5	H51—C30—O9	108.9
H3—C1—N1	109.3	H49—C30—O9	108.7
H2—C1—N1	108.8	H52—C31—O10	107.6
H4—C2—H5	110.2	H52—C31—O13	103.1
H4—C2—H6	109.0	H52—C31—C32	114.4
H4—C2—N1	110.3	H54—C32—H53	109.6
H5—C2—H6	108.4	H54—C32—C31	106.8
H5—C2—N1	110.0	H54—C32—C33	108.1
H6—C2—N1	108.8	H53—C32—C31	106.4
H66—O3—C4	110.0	H53—C32—C33	107.5
H66—O3—H8	111.6	H57—C34—H56	111.2
H7—C3—N1	108.6	H57—C34—H55	109.4
H7—C3—C7	102.0	H57—C34—C33	110.3
H7—C3—C4	112.3	H56—C34—H55	108.4
H8—C4—O3	106.6	H56—C34—C33	109.0
H8—C4—C5	106.2	H55—C34—C33	108.3
H8—C4—C3	107.4	H58—C35—H59	110.7
H8—C4—H66	108.7	H58—C35—H60	108.8
H9—C5—O2	110.6	H58—C35—O11	110.8
H9—C5—O1	106.9	H59—C35—H60	108.8
H9—C5—C4	107.9	H59—C35—O11	110.0
O1—C5—H8	101.8	H60—C35—O11	107.7
H67—O6—C19	111.0	H61—C36—O12	108.6
H10—C6—O1	114.5	H61—C36—C37	112.7
H10—C6—C8	112.5	H61—C36—C33	105.1
H10—C6—C7	109.4	H62—C37—O13	108.1
H68—O7—C21	110.8	H62—C37—C38	109.4
H68—O7—H35	110.4	H62—C37—C36	105.2
H12—C7—H11	109.5	H64—C38—H63	109.3
H12—C7—C6	109.0	H64—C38—H65	109.3
H12—C7—C3	108.7	H64—C38—C37	110.3
H11—C7—C6	108.5	H63—C38—H65	108.9
H11—C7—C3	108.6	H63—C38—C37	109.7
C6—C7—H7	103.0	H65—C38—C37	109.3
H14—C8—H13	109.6	C33—O11—H60	111.0
H14—C8—H15	109.5	H69—O12—C36	109.7
H14—C8—C6	109.5	C36—O12—H70	110.3
H13—C8—H15	109.5	C31—O13—H62	107.2
H13—C8—C6	109.4	H70—O14—H71	104.5

### Hydrogen-bond geometry (Å, º)

**Table:**

*D*—H···*A*	*D*—H	H···*A*	*D*···*A*	*D*—H···*A*
O6—H67···O7	0.83	2.30	2.68 (3)	108
O7—H68···O8	0.82	2.13	2.83 (3)	143
O12—H69···O11	0.83	2.34	2.75 (4)	111
O6—H67···O12^i^	0.83	2.39	2.73 (3)	105
O12—H69···O6^ii^	0.83	2.43	2.73 (3)	102
O14—H70···O12	0.97	1.70	2.65 (4)	168
O14—H71···O7^ii^	0.95	2.58	3.51 (4)	166

Symmetry codes: (i) *x*, *y*, *z*+1; (ii) *x*, *y*, *z*−1.
